# Prevalence of *Echinococcus granulosus sensu lato* in Owned Dogs in Lagos State, Nigeria

**DOI:** 10.3390/vetsci8060101

**Published:** 2021-06-05

**Authors:** Emmanuel Jolaoluwa Awosanya, Zaynab Ligali, Kwabena Obeng Duedu, Angela Peruzzu, Giovanna Masala, Piero Bonelli

**Affiliations:** 1 Department of Veterinary Public Health and Preventive Medicine, University of Ibadan, Ibadan PMB 20005, Nigeria; zuwaynibl@gmail.com; 2 Department of Biomedical Sciences, School of Basic and Biomedical Sciences, University of Health and Allied Sciences, Ho PMB 31, Ghana; kduedu@uhas.edu.gh; 3 OIE Reference Laboratory for Echinococcosis, National Reference Laboratory for Cystic Echinococcosis (CeNRE), Istituto Zooprofilattico Sperimentale della Sardegna (IZS), 07100 Sassari, Italy; angela.peruzzu@izs-sardegna.it (A.P.); giovanna.masala@izs-sardegna.it (G.M.)

**Keywords:** *Echinococcus granulosus sensu lato*, dogs, Nigeria

## Abstract

*Echinococcus granulosus sensu lato (s.l.)* infection in dogs poses risk of transmission to their owners and family members. We determined the prevalence and factors associated with *E. granulosus s.l.* infection among owned dogs presented at veterinary clinics or hospitals in Lagos State, Nigeria. Fecal samples from 217 dogs were screened for the presence of taeniid eggs using a sedimentation test in a cross sectional study. The taeniid eggs were identified at molecular level using a multiplex PCR. A structured questionnaire was used to obtain data on intrinsic and extrinsic factors from 133 dog owners. Out of the 217 dog fecal samples, 13 (6.0%) had taeniid eggs, of which 12 (92.3%) were identified as *Echinococcus granulosus s.l.* We found that *Echinococcus granulosus* infection is present among owned dogs in Lagos State with an overall prevalence of 5.5%. Location of the veterinary clinics or hospital and purpose for keeping dogs were significant factors associated with *E. granulosus* infection among owned dogs. Dogs living in suburban areas and kept for security purposes or guarding have higher probability of infection. Appropriate and regular treatment of dogs with praziquantel is highly recommended to reduce risk of *E. granulosus* transmission to humans.

## 1. Introduction

*Echinococcus granulosus sensu lato* (*s.l.*) is a flatworm of the class *Cestoda* family *Taeniidae* comprising five different species (*Echinococcus sensu stricto*, *Echinococcus ortleppi*, *Echinococcus equinus*, *Echinococcus canadensis*, *Echinococcus felidis*) [1,2]. The larval stage of *E. granulosus s.l.* is considered the etiological agent of cystic echinococcosis (CE). The strong zoonotic potential of *E. granulosus s.l.*, especially related to *E. granulosus s.s.* [3] infection, is cause of great concern for public health worldwide. CE is distributed in every continent except Antarctica, and in endemic regions (southern and eastern Europe, southern America, central Asia, China, and northern and eastern Africa) the prevalence of CE in humans could be as high as between 5% to 10% [4]. Considering its consistent clinical and economic burden, the World Health Organization (WHO) included CE in a list of seven neglected zoonotic diseases requiring priority intervention.

The life cycle of *E. granulosus s.l.* is indirect and includes definitive (wild and domestic carnivores) and intermediate hosts (wild and domestic ungulates). The adult parasite lives in the small intestine of the definitive host responsible of eggs dispersion with the feces. Intermediate hosts are infected through the ingestion of the eggs that develop in the internal organs into the larval form (metacestode). Accidentally, humans can acquire infection, acting as an aberrant host. The life cycle continues when definitive hosts feed on infected organs of intermediate hosts. Conversely, the aberrant hosts represent an epidemiological dead end [5]. 

Domestic dogs have a prominent role in the transmission of *E. granulosus* infection to humans either directly or indirectly through environmental contamination of soil, water, or vegetables. Several studies confirmed the epidemiological importance of owned and stray dogs, revealing extremely varied values of prevalence rates in the African continent ranging from 12.2% to 51.3% [6,7,8,9]. CE is highly endemic in sub-Saharan Africa, but to date the picture of the epidemiological situation for *E. granulosus* in this area is still incomplete [10]. Despite the differences in prevalence of CE among the various countries, a common pattern related to livestock farming, slaughter hygiene, presence of free-roaming dogs, and socio-cultural aspects, can be identified throughout sub-Saharan Africa [11]. Particularly, dog-ownership, as well as the uncontrolled access of dogs to slaughter facilities, have been associated to a significant risk of *E. granulosus s.l.* infection [7,12]. 

In Nigeria, the serological evidence of CE in humans (3.3%) has been reported in Plateau State [13]. Likewise, a seroprevalence study carried out in the southwest of Nigeria, showed a high value of prevalence of *E. granulosus* infection among dogs (12.45%) [14]. More so, the occurrence of hydatid cysts during meat inspection in livestock animals has also been reported in slaughter facilities in Nigeria [15,16,17,18]. These findings attest to the public health challenge of CE in humans and animals in Nigeria. For the concurrent presence of one of the largest slaughter facilities in Nigeria, with daily throughput of about 3000 livestock [19,20] and of other conditions regarded as risk factors for human CE, we focused our investigation on the Lagos State area. Considering the limited amount of data on the epidemiology of *E. granulosus s.l.* in Nigeria, we carried out this study to determine the prevalence of the infection and its associated factors among owned dogs. Furthermore, identification of risk factors for *E. granulosus* infection in dogs will aid informed control and prevention strategies in dogs and guide to reduce or prevent risk of infection in humans. 

## 2. Materials and Methods

### 2.1. Study Design

**Figure 1:**
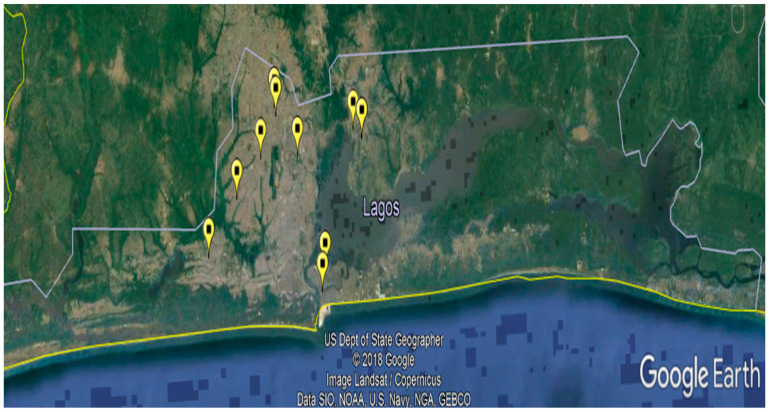
The map of Lagos State, Nigeria showing the locations of the public and private owned veterinary clinics and hospitals involved in the study.

This was a cross sectional study carried out between September 2018 and March 2019. The study area was Lagos State, southwest Nigeria. Lagos State is a metropolitan city considered the economic capital of Nigeria. It is approximately between longitude 3°10’0.00” E and 3°60’0.00” E and between latitude 6°42’0.00” N and 6°70’0.00” N. The state borders with Ogun State to the North and the East, with Republic of Benin to the West, and stretches over 180 km along the Guinea Coast of the Bight of Benin on the Atlantic Ocean. The projected human population for 2016 was 24,051,762 [21]; the dog population within compound was estimated at 1447, constituting half (50.3%) of the total dog population with a dog to human ratio of 1:5.6 [22]. Lagos State has five administrative divisions: Ikeja, Lagos Island, Badagry, Ikorodu, and Epe. Ikeja and Lagos Island divisions have mostly towns and cities, and scarcely villages; while Badagry, Ikorodu, and Epe divisions have a substantial presence of villages and towns. The study locations were registered public and private veterinary clinics or hospitals in four of the administrative divisions namely Ikeja, Lagos Island, Badagry, and Ikorodu: two each of urban and semi-urban settings. Five, two, one, and two recognized veterinary clinics or hospitals were involved in the study from Ikeja, Lagos Island, Badagry, and Ikorodu administrative divisions of the state, respectively (Figure 1).

The owned dogs presented for routine clinical check-up, about 64.0% of owned dogs [22], constituted our target population. Pregnant bitches and puppies below the age of four weeks were excluded from the study because the praziquantel used to aid taeniid egg expulsion in the study is contraindicated in these animals. All the dogs whose owner consented to participate in the study were enrolled. 

### 2.2. Sample Size and Sampling

We calculated the sample size (*n*) for the dogs using the formula [23]

*n* = (Z^2^ p (1−p))⁄d^2^, (1)

where Z is the reliability coefficient 1.96 at 95% confidence level; p is the prevalence of taeniid eggs in dogs at 4.86% (unpublished work); d is the precision at 5%.

After adjusting for 10% non-response, we obtained a sample size of 217 dogs. Purposive sampling was used to select the public or private veterinary practices involved in the study from each division of the state based on their clientele. A total of 10 veterinary clinics or hospitals were selected. We sampled an average of 20 dogs per clinic or hospital, the actual number of dogs screened was however modified by the clientele base of each participating clinic or hospital. All dogs presented at each veterinary clinic or hospital were sampled until the calculated sample size was reached.

### 2.3. Fecal Samples Collection

Praziquantel was administered at 5 mg/kg per os to all participating dogs in order to improve taeniid egg detection by fecal examination [24]. About 10 g of fecal samples were collected 12–24 h post praziquantel administration either by the veterinarian during the clinical visit, per rectum, or by the owners, off the ground. The fecal samples obtained were labelled corresponding to the unique identifier on the questionnaire administered to their owners. The Ziploc bags containing the fecal samples were sent on ice-packs in coolers to the laboratory at the Department of Veterinary Public Health and Preventive Medicine, University of Ibadan and stored at −20 °C until use.

### 2.4. Data Collection

Structured questionnaire was used to obtain data on the demography of the owners; intrinsic factor of dogs such as age, sex, and breed; and extrinsic factors which include management and environmental factors.

### 2.5. Copromicroscopic and Molecular Analyses

A modification of the formalin-ether sedimentation technique [25,26] was used for the taeniid egg detection in fecal samples. Briefly, about 1 g of stool was collected with a spatula and emulsified in 3 mL of 10% formalin in a mortar using a pestle for proper emulsification. The stool emulsion was poured through a fine mesh of 250 µm into a 15 mL centrifuge tube. The stool was washed through the gauze using 2 mL of 10% formalin. About 3 mL of ethyl acetate, in substitution of diethyl ether of the original method [27], was added to the content of the centrifuge tube and mixed. The mixture was centrifuged at 448 g for 5 min. After centrifuging, four layers were formed in the tube; an ethyl acetate layer, plug of debris, formalin layer and the sediment. The top three layers were discarded and the sediment was mixed thoroughly. Two drops of sediment were placed on a glass slide with one drop of iodine solution and a cover slip was applied. The entire coverslip was then examined under an optical microscope (Olympus, Tokyo, Japan) (4×, 10× and 40×). 

The detected taeniid eggs were further identified by molecular analyses. Genomic DNA was extracted using the QIAamp^®^ Fast DNA Stool Mini Kit (Qiagen, Hilden, Germany), and a multiplex polymerase chain reaction protocol was performed as already described [28] at the Istituto Zooprofilattico Sperimentale (IZS) della Sardegna, Italy. The total reaction mix volume was 25 µL: 12.5 µL Master Mix (Quanti-Tect Probe PCR Master Mix-QIAGEN^®^); 0.5 µL of Primer Cest 1, 2, 3, 4 (2 pmol/µL) and of Primer Cest 5 (16 pmol/µL); 5 µL nuclease-free water and 5 µL of template DNA (5 ng DNA per tube). Primers amplify fragments of the mitochondrial NADH dehydrogenase subunit 1 gene (*nad1*) specific for *E. multilocularis* (Cest 1and 2), cyto-chrome oxidase subunit 1 (*cox1*) gene specific for *E. granulosus s.l.* (Cest 3 and 5) and the small sub-unit of ribosomal RNA (*rrnS*) specific for *Taenia* spp. [28]. *E. granulosus s.l.* reference positive DNA produced by IZS della Sardegna 10 ng (5 µL) was used as PCR positive control while sterile water as a negative control. Agarose gel electrophoresis was performed on precast gels (E-Gel™ EX Agarose Gels, 2% Agarose, Thermo Fisher, Waltham, MA, USA) using the E-Gel™ Power Snap Electrophoresis System (Thermo Fisher, Waltham, MA, USA). DNA ladder (Marker VIII, Merk Life Science, Darmstadt, Germany) was loaded onto the agarose gel for size determination of the PCR products.

### 2.6. Statistical Analysis

All data were entered into Microsoft Excel^®^ 2010 and analyzed using Epi Info^®^ version 7.2.4.0. The prevalence (P) of dogs harboring taeniid eggs or *Echinococcus granulosus* eggs was determined using the formula:

P (%) = (n/N) × 100,(2)

where n is the number of taeniid eggs or *E. granulosus* infected dogs and N is the total number of dogs screened. 

The outcome variables in the bivariate analyses are the number of *E granulosus* infected owned dogs and the proportion of owners with *E. granulosus* infected dogs. Frequencies, percentages, means and standard deviations were determined. We also determined odds ratios (OR) and the 95% confidence intervals (CI) in order to assess association between the outcome variables and the independent variables such as demographic characteristics, intrinsic and extrinsic factors. The level of significance in the differences observed was determined using the Fisher’s exact test at 5% level of significance.

## 3. Results

The present data are the results of a cross sectional study carried out in Lagos State, southwest Nigeria, between September 2018 and March 2019. The prevalence of *E. granulosus s.l.* was estimated in the owned dogs of four administrative divisions: two urban, Ikeja and Lagos Island, and two sub-urban areas, Badagry and Ikorodu. Fecal samples were collected from a representative number (*n* = 217) of adult dogs presenting at veterinary practices in order to detect the presence of *E. granulosus s.l.* A questionnaire was administered to dog owners to find factors associated with the ownership that are more likely to be related to CE.

### 3.1. Socio-Demographic Variables of Dog Owners and Dogs Characteristc

The mean age of the dog owners was 39.1 ± 11.4 years. Out of the 133 dog owners, 97 (72.9%) were male. The majority (81.2%) were tertiary-educated, employed (87.2%), married (69.9%), and belonging to Yoruba ethnic group (54.9%) (Table 1). The median age of the dogs screened was 24.5 months (interquartile range 10.0–39.0). Out of the 217 dogs screened, 209 (96.3%) were exotic breed of which the Alsatian was the more represented (22.1%). More than half (53.9%) of the dogs were male (Table 2).

### 3.2. E. granulosus s.l. Infection and Associated Factors in Dogs

**Figure 2:**
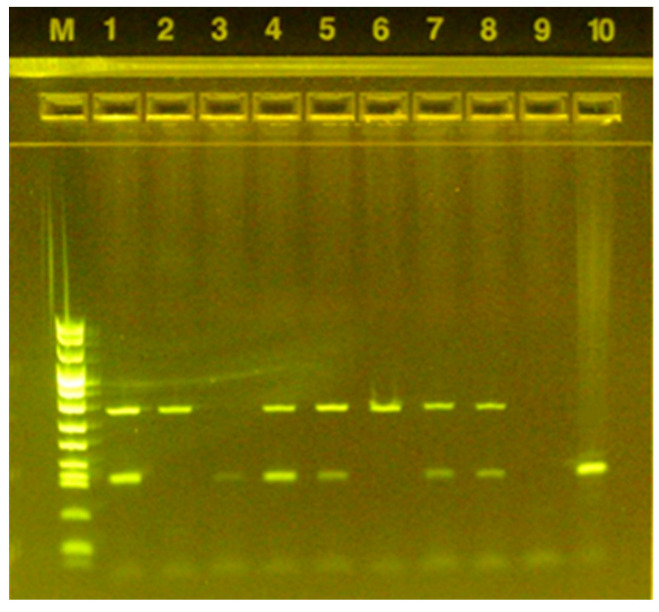
Agarose gel electrophoresis following multiplex PCR amplification of DNA samples extracted from taeniid eggs. Lane M: marker; lanes 2 and 6: *Taenia spp.* positive samples; lane 3 *E. granulosus s.l.* positive sample; lanes 1, 4, 5, 7, and 8: samples with mixed infection (*Taenia spp.* and *E. granulosus s.l.*); lane 9: negative control; lane 10: positive control.

Out of the 217 dogs screened, owned by 133 persons, taeniid eggs were seen in 13 dogs (6.0%, CI: 2.8–9.2%), of which 12 (92.3%, CI: 77.8–100.0%) were found to be infected by *E. granulosus s.l.* and 10 (76.9%, CI: 54.0–99.8%) had mixed infection of both *E. granulosus s.l.* and *Taenia spp. Echinococcus multilocularis* was not detected in any of the taeniid eggs tested. Out of the 133 dog owners, 11 (8.3%, CI: 3.6–13.0%) had at least one dog infected with *E granulosus s.l.* A representative electrophoresis gel image of the *E. granulosus s.l.* positive samples is shown in Figure 2. 

The overall *E. granulosus s.l.* prevalence was 5.5% (12 out of 217). The prevalence of *E. granulosus s.l.* in females (7.0%, CI: 2.0–12.0%) was higher than in male dogs (4.3%, CI: 0.6–7.9%). Dogs aged more than 12 months had higher (6.0%, CI 2.0–10.0%) *E. granulosus s.l.* infection rate than those aged 12 months or below (4.8%, CI: 0.2–9.4%). Higher *E. granulosus s.l.* prevalence was also found in the local breed of dogs (12.5%, CI: 0.0–35.4%) than in the exotic (5.3%, CI: 2.2–8.3%). However, none of these intrinsic factors were significantly associated with *E. granulosus s.l.* infection (Table 3). Dogs screened at Badagry had the highest *E. granulosus s.l.* infection rate of 22.5% (CI: 9.6–35.4%), followed by Ikorodu (4.35%, CI: 0.0–12.2%) and Lagos Island (2%, CI: 0.0–5.8%) while those from Ikeja had the least infection rate (1.0%, 0.0–2.9%). Dogs screened at veterinary clinics or hospitals in Badagry were significantly 14 and 25 times more likely to be *E. granulosus s.l.* infected when compared to those from Lagos Island and Ikeja, respectively (Table 3). 

Dog owners who reported using their dogs solely for security purpose contributed most (19.0%, CI: 7.2–30.9%) to the population of owners of *E. granulosus s.l.* infected dogs, while those who reported using their dogs for breeding and other purposes contributed the least (2.4%, CI: 0.0–7.0%) (Table 4). Dog owners who use their dogs for breeding and other purposes were significantly less likely to have *E. granulosus s.l.* infected dogs when compared to those who used their dogs solely for security purposes. More so, those whose sole purpose of owning their dogs was for companionship were less likely to have *E. granulosus s.l.* infected dogs compared to those whose purpose was solely for security (Table 4). The owners involved in this study administered routinely anthelminticsto their dogs. Dogs treated with praziquantel turned out to have a lower *E. granulosus s.l.* infection rate (2.0%, CI: 0.0–6.0%) compared to those treated with other anthelmintics (13.5%, CI: 2.5–24.5%). However, the type of anthelmintic used was not significantly (*p* > 0.05) associated with owning *E. granulosus s.l.* infected dogs. Other extrinsic factors which included sociodemographic, management, and environmental variables such as dog owners’ characteristics, number of dogs owned, allowing dog to roam about, feeding of raw meat, feeding of offal, having slaughter slabs or abattoir in the vicinity, frequency of deworming, water source, and feed types were not significantly associated with owning an *E. granulosus s.l.* infected dog (Table 4). We did attempt a multivariable logistic regression but only one variable as in the bivariate remained significant adjusting for other co-variates at *p* < 0.25 in the model. For this reason we decided not to include the results.

## 4. Discussion

The present study aimed to determine the *E. granulosus s.l.* prevalence among owned dogs in Southwest Nigeria. To this purpose fecal samples were collected from 217 dogs presented at veterinary clinics or hospitals located in several divisions of Lagos State. Microscopic and molecular analyses were performed to detect the presence of *E. granulosus s.l.* Our results showed that the *E. granulosus s.l.* prevalence in the target population was 5.5%, which is similar to what was observed in Kosovo [29]. Higher prevalence was reported in the same target population in Uganda [7], Libya [30], China [12], Turkey [31], and Morocco [6], whereas no evidence of *E. granulosus* was detected among owned dogs in Germany and other European countries [32]. The *E. granulosus* prevalence in dogs that we observed in Nigeria is in agreement with a recent meta-analysis study that reported markedly lower values in West Africa (5.5%) compared to East (23.4%) and North Africa (24.7%) [33]. The owners’ perception about dog welfare together with the awareness that companion animal and human health are interconnected represent a fundamental prerequisite for the prevention of *E. granulosus* infection. The *E. granulosus s.l.* prevalence value observed in the present study could have been affected by the veterinary care given to the owned dogs and especially by the anthelmintic treatment that all the owners reported to regularly administered to their animals. It should also be noted that the treatment effectiveness is improved with proper deworming of dogs with praziquantel at appropriate interval [6]. This study offers a picture of a specific subpopulation of dogs that can benefit from high standards of health and welfare but it also give us a reason to possibly expect a higher prevalence in the overall dog population in Lagos State, if less privileged dogs, such as stray and rural dogs, were included.

The location of the veterinary clinics or hospitals were associated with different likelihood of *E. granulosus s.l.* infection among owned dogs. In this study the prevalence of *E. granulosus s.l.* infection was significantly higher in Badagry compared to Lagos Island and Ikeja. No significant differences were found between Badagry and Ikorodu, both sharing the same social and cultural rural context. Human settlements in these latter divisions of Lagos State are characterized by small communities and villages as opposite to densely populated cities that can be found in Ikeja and Lagos Island. Indeed Lagos Island and Ikeja, which is the state capital, have a higher level of urbanization that ensures higher standards of hygiene and biosafety. Some epidemiological factors, such as owners’ poor health education, indicators of poverty, possibility of scavenging dead animals, and lack of adequate anthelmintic treatment that have been reported to be associated with *E. granulosus* infection in dogs [34], may be more prevalent in areas characterized by a low level of urbanization. Likewise, the uncontrolled slaughtering of livestock animals and the presence of grazing livestock animals, more easily encountered in rural areas, have been related with an increased *E. granulosus* prevalence in dogs [7,29]. This calls for concerted efforts towards the prevention and control of *E. granulosus* infection among owned dogs especially in less urbanized areas. 

In agreement with other authors [12,14,31,35] our results indicated that there is no significant association between the age and sex of dogs and the observed prevalence of *E. granulosus*. Conversely, we found that the prevalence of *E. granulosus* was significantly higher in guard dogs than in other categories examined in this study. Guard dogs in rural villages have higher likelihood of scavenging on viscera harboring viable CE cysts, thus allowing the parasite life cycle completion [34]. Furthermore, as already suggested by Liu et al. [12], the tendency to underfeed dogs in rural areas or to provide them poor diet quality encourages their predatory behavior thus increasing the risk of acquiring *Echinococcus* infection. In this respect, rodents have been assumed to play a role in the transmission of *E. granulosus* acting as intermediate hosts potentially preyed by dogs. Several authors reported this eventuality in North Africa, especially in urban and suburban areas [33,36], but its possible role in *E. granulosus* transmission to dogs in Nigeria need to be further investigated. Other extrinsic factors, such as dog owners’ characteristics, number of dogs owned in the household, dog roaming, having slaughter slabs in the vicinity, feeding of visceral or offal, and dog deworming frequency, were not significantly associated with *E. granulosus* infection in dogs in this study. This is also similar to the findings of Liu et al. [12] among owned dogs in China. The influence of some of the extrinsic factors could have been biased by the deworming status of the dogs. Although, there is no significant association between the administration of anthelmintics and *E. granulosus* infection, it should be noted that dogs usually treated with praziquantel by their owners had the least prevalence. 

This study has some limitations in that there could be a recall bias in some of the responses of the dog owners to questions on the extrinsic factors. This was mitigated by triangulation via repeating some questions differently. In addition, we could not determine the genotype of *E. granulosus* eggs samples examined in this study. Unfortunately, DNA extraction from formalin-fixed parasite eggs did not provide samples of adequate quality for nucleotide sequencing. Further studies are needed to analyze genetic diversity in Nigeria and to improve our knowledge on the epidemiology of the parasite aiming at the development of more effective measures for prevention and control of *E. granulosus s.l.* Lastly, we did not use any purgative, such as arecoline, which could have further aided the expulsion of the taeniid cestode or the eggs; however, the use of praziquantel before feces collection was useful in approximating the true prevalence of *E. granulosus* infection.

## 5. Conclusions

In comparison to other studies from African countries, a lower prevalence of *Echinococcus granulosus s.l.* among owned dogs was found in Lagos State. Dogs living in suburban areas and kept for security purposes were more likely to become infected. Appropriate and regular treatment of praziquantel performed by qualified personnel is highly recommended to reduce risk of *E. granulosus* transmission to humans.

## Figures and Tables

**Table 1:** Socio-demographic characteristics of the dogs owners (*n* = 133) presented at veterinary clinics or hospitals in Lagos State, Southwest Nigeria, in 2018–2019.

Variables	Characteristics	Frequency	Percentage (95% CI)
Age (Years)	17–35	59	44.4 (35.9–52.8)
	>35	74	55.6 (47.2–64.1)
Gender	Male	97	72.9 (65.4–80.5)
	Female	36	27.1 (19.5–34.6)
Ethnic origin	Yoruba	73	54.9 (46.4–63.3)
	Igbo	33	24.8 (17.5–32.2)
	Hausa	2	1.5 (0.0–3.6)
	Others ^1^	25	18.8 (12.2–25.4)
Highest educational level	No formal education	1	0.8 (0.0–2.2)
	Primary	0	0
	Secondary	24	18.1 (11.5–24.6)
	Tertiary	108	81.2 (74.6–87.8)
Marital status	Single	40	30.1 (22.3–37.9)
	Married	93	69.9 (62.1–77.7)
Occupational status	Unemployed	17	12.8 (7.1–18.5)
	Employed	54	40.6 (32.2–49.0)
	Self employed	62	46.6 (38.1–55.1)

^1^ Other ethnic groups included Urhobo and Bini. CI = Confidence Interval.

**Table 2:** Dogs characteristics (*n* = 217) presented at veterinary clinics or hospitals in Lagos State, Southwest Nigeria, in 2018–2019.

Variables	Characteristics	Frequency	Percentage (95%) CI
Age (months)	2–12	83	38.3 (31.8–44.7)
	>12	134	61.7 (55.3–68.2)
Sex	Male	117	53.9 (47.3–60.6)
	Female	100	46.1 (39.5–52.7)
Breed	Alsatian	48	22.1 (16.6–27.6)
	Lhasa Apso	28	12.9 (8.4–17.4)
	Boerboel	25	11.5 (7.3–15.8)
	Caucasian	25	11.5 (7.3–15.8)
	Rottweiler	19	8.8 (5.0–12.5)
	Terrier	10	4.6 (1.8–7.4)
	Pit bull	9	4.2 (1.5–6.8)
	Others ^1^	45	20.7 (15.3–26.1)
	Local	8	3.7 (1.2–6.2)
Location	Ikeja	102	47.0 (40.4–53.6)
	Lagos Island	51	23.5 (17.9–29.1)
	Badagry	40	18.4 (13.3–23.6)
	Ikorodu	24	11.1 (6.9–15.2)

^1^ Other breed of dogs included Samoyed, Maltese, Poodle, Saint Bernard, Cane Corso, American Eskimo, Great Dane, Labrador Retriever, Weimaraner, Shih Tzu, Japanese Tosa, Pomeranian, Pekingese, Labradoodle, Golden Doodle, French Mastiff, Bullmastiff, Doberman Pinscher, Dalmatian, Chihuahua and Basenji. CI = Confidence Interval.

**Table 3:** Intrinsic and extrinsic factors associated with *Echinococcus granulosus s.l.* (*E.g. s.l.*) infection among owned dogs (*n* = 217) presented at veterinary clinics or hospitals in Lagos State, Southwest Nigeria, in 2018–2019.

Variables	Characteristics	*E.g. s.l.* Positive *n* = 12 (%)	*E.g. s.l.* Negative *n* = 205 (%)	Odds Ratio OR, (95% CI)	*p* Value
Age (months)	2–12	4 (33.3)	79 (38.5)	0.80 (0.17, 3.10)	1.00
	>12	8 (66.7)	126 (61.5)		
Sex	Male	5 (41.7)	112 (54.6)	0.59 (0.14, 2.26)	0.55
	Female	7 (58.3)	93 (45.4)		
Breed	Local	1 (8.3)	7 (3.4)	2.56 (0.05, 23.08)	0.37
	Exotic	11 (91.7)	198 (96.6)		
Location	Badagry	9 (75)	31 (15.1)	Ref.	
	Ikorodu	1 (8.3)	23 (11.2)	0.15 (0.003, 1.24)	0.08
	Lagos Island	1 (8.3)	50 (24.4)	0.07 (0.002, 0.55)	0.004 *
	Ikeja	1 (8.3)	101 (49.3)	0.04 (0.001, 0.27)	<0.001 *

* Significant at *p* < 0.05; CI = Confidence interval.

**Table 4:** Extrinsic factors associated with owning *Echinococcus granulosus s.l.* (*E.g. s.l.*) infected dogs in Lagos State, Southwest Nigeria, 2019 (*n* = 133).

Variables	Characteristics	Owners of *E.g. s.l.* Positive Dogs *n* = 11 (%)	Owners of *E.g. s.l.* Negative Dogs *n* = 122 (%)	Odds Ratio OR, (95% CI)	*p* Value
**Dog Owners’ Characteristics**					
Age (Years)	14–35	5 (45.5)	54 (44.3)	1.05 (0.24, 4.38)	1.00
	>35	6	68		
Gender	Male	10 (90.9)	87 (71.3)	3.99 (0.53, 179.50)	0.29
	Female	1	35		
Ethnic origin	Yoruba	3 (27.3)	70 (57.4)	0.28 (0.05, 1.24)	0.07
	^#^ Others	8	52		
Highest educational level	Secondary and below	3 (27.3)	22 (18.0)	1.70 (0.27, 7.83)	0.43
	Tertiary	8	100		
Marital status	Single	4 (36.4)	36 (29.5)	1.36 (0.28, 5.76)	0.73
	Married	7	86		
Occupational status	Unemployed	3 (27.3)	14 (11.5)	2.86 (0.44, 13.87)	0.15
	Employed	8	108		
**Management and Environmental Factors**					
Number of dogs in household	1	8 (72.7)	71 (58.2)	1.91 (0.43, 11.70)	0.52
	>1	3	51		
Allow dog to roam	Yes	1 (9.1)	40 (32.8)	0.21 (0.01, 1.54)	0.17
	No	10	82		
Purpose for keeping dog	Security	8	34	Ref.	
	Companion	2 (18.2)	47 (38.5)	0.18 (0.02, 1.00)	0.05 *
	Breeding and others	1 (9.1)	41 (33.6)	0.11 (0.00, 0.86)	0.03 *
Feed raw meat to dog	Yes	2 (18.2)	13 (10.7)	1.85 (0.18, 10.48)	0.36
	No	9	109		
Feed offal to dog	Yes	2 (18.2)	29 (23.8)	0.71 (0.07, 3.7)	1.00
	No	9	93		
Slaughter slab in the vicinity	Yes	1 (9.1)	16 (13.1)	0.66 (0.01, 5.28)	1.00
	No	10	106		
Dewormed dog	Yes	11 (100.0)	122 (100.)	∞ (∞, ∞)	∞
	No	0	0		
Frequency of deworming	Once a month	2	31	Ref	
	4 times a year	6 (54.6)	37 (30.3)	2.49 (0.41, 26.89)	0.47
	When necessary	3 (27.3)	54 (44.3)	0.86 (0.09, 10.85)	1.00
Use of anthelmintic	Praziquantel	1	48	Ref.	
	Other drugs	5 (45.5)	32 (26.2)	7.34 (0.77, 361.90)	0.10
	Unknown	5 (45.5)	42 (34.4)	5.63 (0.60, 275.80)	0.19
Source of water	Borehole	10	77	Ref.	
	Tap	1 (9.1)	33 (27.1)	0.24 (0.01, 1.78)	0.26
	Deep well	0 (0.0)	12 (9.8)	0.00 (0.1, 3.33)	0.51
Type of food	Home-made only	4	27	Ref.	
	Canned food only	1 (9.1)	17 (13.9)	0.40 (0.01, 4.55)	0.77
	Home-made and canned	5 (45.5)	61 (50.0)	0.56 (0.11, 3.04)	0.62
	Others	1 (9.1)	17 (13.9)	0.40 (0.01, 4.55)	0.77

* Significant at *p* < 0.05; CI = Confidence interval; ^#^ Other ethnic groups included Igbo, Hausa, Urhobo and Bini; ∞ = undefined.

## Data Availability

The data presented in this study are available within the article.
